# Mandibular Osseous Changes in Children and Adolescents With Beta Thalassemia Major: A Panoramic Radiomorphometric Study

**DOI:** 10.7759/cureus.114309

**Published:** 2026-08-10

**Authors:** Himabindu S, Narjish Rahman Barbhuiya, Spurthi Raikote, Chandrasekhar M Reddy, Sivan Sathish

**Affiliations:** 1 Department of Oral Medicine and Radiology, Government Dental College and Hospital, Rajiv Gandhi Institute of Medical Sciences (RIMS) University, Kadapa, IND; 2 Department of Oral Medicine and Radiology, Teerthanker Mahaveer Dental College and Research Centre, Teerthanker Mahaveer University, Moradabad, IND

**Keywords:** beta thalassemia, bone density, bone remodelling, mandible, panoramic radiography

## Abstract

Background: Beta thalassemia major is an inherited hemoglobin disorder characterized by deficient β-globin production, chronic anemia, and expansion of hematopoietic marrow. Prolonged marrow activity can alter craniofacial bone architecture and reduce mandibular cortical integrity.

Objective: This study compared mandibular cortical characteristics in children and adolescents with beta thalassemia major and matched healthy participants using panoramic radiomorphometric measurements.

Methods: Sixty participants aged 8-19 years were included: 30 individuals with beta thalassemia major and 30 age- and sex-matched controls. Digital panoramic radiographs were examined using the Mental Index, Panoramic Mandibular Index, Antegonial Index, Gonial Index, and Mandibular Cortical Index. Repeat measurements were used to determine intraobserver agreement, and intergroup differences were assessed using appropriate statistical tests.

Results: The thalassemia group showed lower values for all quantitative mandibular cortical indices than the control group. Eroded C2 and C3 cortical patterns were also more frequent among affected participants, indicating reduced cortical integrity and increased endosteal remodeling.

Conclusion: Panoramic radiomorphometric assessment can identify mandibular cortical alterations in young patients with beta thalassemia major and may support opportunistic screening during routine dental imaging.

## Introduction

Beta thalassemia major results from markedly reduced or absent production of the β-globin chain. The resulting ineffective erythropoiesis and chronic anemia usually necessitate repeated transfusion therapy. When erythropoietic activity remains increased, marrow spaces may enlarge within several skeletal regions, including the maxillofacial complex. Progressive expansion can alter trabecular organization, reduce cortical thickness, and contribute to craniofacial deformity [1-3].

Radiographic changes associated with the disorder may include coarsening of trabeculae, thinning of cortical boundaries, enlargement of diploic spaces, and a hair-on-end appearance of the skull [4]. Detecting these changes during childhood is clinically relevant because skeletal development is still ongoing. Digital panoramic radiography provides broad visualization of the jaws with relatively low radiation exposure and is commonly available in dental practice, making it suitable for evaluating mandibular changes [5].

Mandibular radiomorphometric indices provide objective measurements of cortical dimensions at defined anatomical sites. The Mental Index (MI) and Panoramic Mandibular Index (PMI) assess the cortex near the mental foramen, whereas the Antegonial Index (AI) and Gonial Index (GI) assess cortical thickness in posterior mandibular regions [6,7]. The Mandibular Cortical Index (MCI) supplements these measurements by grading the appearance of the endosteal cortical margin [8-10]. These parameters have previously been used to identify mandibular changes associated with systemic skeletal disorders.

Evidence regarding these indices in pediatric beta thalassemia remains limited. Published studies have generally included relatively small samples and have not consistently combined quantitative cortical measurements with qualitative assessment of cortical erosion. A recent systematic review also identified insufficient evidence regarding mandibular imaging changes in young patients with thalassemia [11].

As childhood and adolescence are critical periods for skeletal maturation, early identification of mandibular cortical thinning and altered cortical morphology may improve dental risk assessment and facilitate timely referral for further skeletal evaluation. Recognition of these changes during routine dental imaging may also support multidisciplinary medical-dental management by alerting clinicians to compromised bone health before invasive dental procedures, orthodontic treatment, or other interventions are undertaken. This study therefore compared MI, PMI, AI, GI, and MCI between children and adolescents with beta thalassemia major and matched healthy controls.

## Materials and methods

Study design

A comparative cross-sectional investigation was undertaken using digital panoramic radiographs from children and adolescents with beta thalassemia major and matched systemically healthy controls. The Institutional Ethics Committee approved the study protocol. The investigation followed the ethical requirements of the Declaration of Helsinki. Written permission was obtained from parents or legal guardians, and participant information was handled confidentially.

Study population

The required sample size was calculated a priori using GPower software (version 3.1, Heinrich Heine University Düsseldorf, Düsseldorf, Germany). The calculation was based on a two-sided independent-samples t-test, an alpha level of 0.05, statistical power of 80%, and an effect size derived from previously published studies comparing panoramic mandibular radiomorphometric indices between patients with beta thalassemia major and healthy controls. A minimum sample of 60 participants, comprising 30 cases and 30 controls, was considered sufficient to detect statistically significant intergroup differences [12].

The case group consisted of transfusion-dependent patients with confirmed beta thalassemia major who were receiving regular care at a thalassemia center in South India. The comparison group included age- and sex-matched children and adolescents without known systemic conditions affecting bone metabolism.

Participants were eligible if they were between 8 and 19 years of age. Cases were required to have a confirmed diagnosis of transfusion-dependent beta thalassemia major and to be undergoing regular blood transfusion therapy, whereas controls were required to be systemically healthy without any known disorders affecting bone metabolism. Participants were excluded if they had thalassemia intermedia or other hemoglobinopathies, systemic diseases known to influence bone metabolism, endocrine disorders such as diabetes mellitus, hypothyroidism, or hypoparathyroidism, cardiac or hepatic complications, a history of mandibular trauma or surgical intervention, or panoramic radiographs with positioning errors, artifacts, or inadequate diagnostic quality.

Image acquisition and analysis

Digital panoramic radiographs were obtained using a standardized digital panoramic imaging system (Vatech PCH-30CS, Vatech Co., Ltd., Hwaseong, South Korea). Images were acquired using standardized exposure parameters of 67 kVp, 9 mA, 11.5 seconds exposure time, and 12.1 seconds scan time. During image acquisition, participants were positioned according to the manufacturer's recommendations. The Frankfort horizontal plane was oriented parallel to the floor, the midsagittal plane was maintained perpendicular to the floor, and the maxillary and mandibular incisors were positioned in the bite block groove to ensure standardized head positioning and minimize geometric distortion and magnification variations. All radiographs were exported and analyzed using EzDent-i digital imaging software (Vatech Co., Ltd., South Korea).

Radio-morphometric assessment

**Figure 1:**
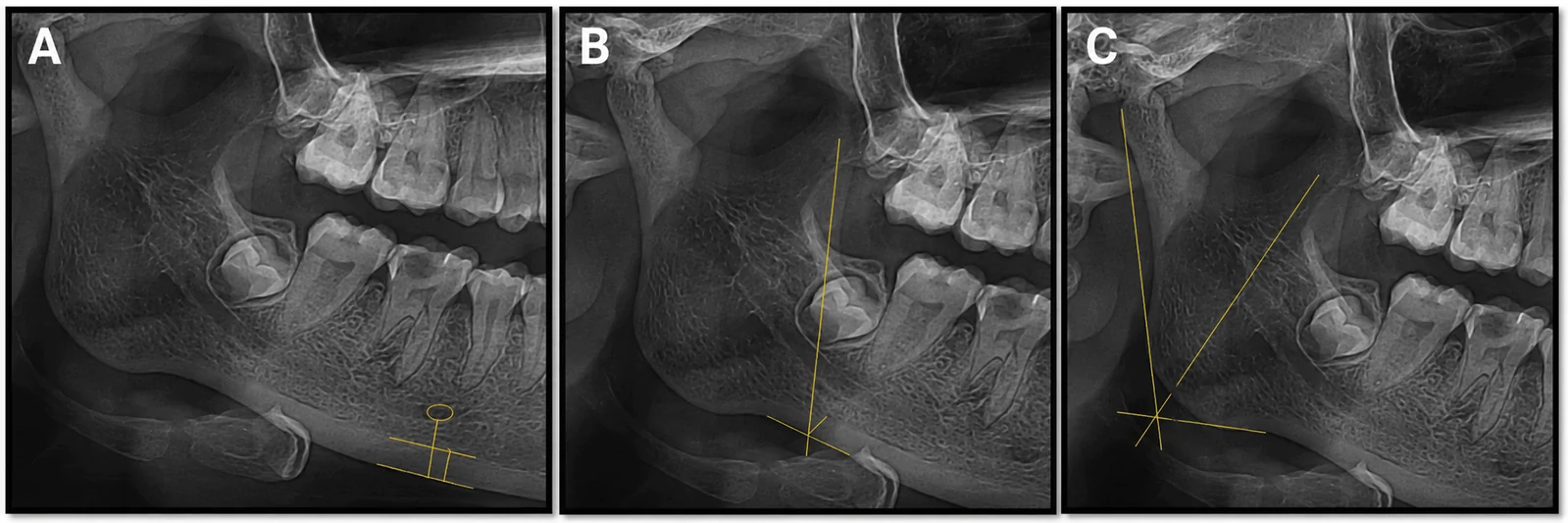
Panoramic mandibular radiomorphometric indices. (A) Panoramic Mandibular Index (PMI). (B) Antegonial Index (AI). (C) Gonial Index (GI).

**Figure 2:**
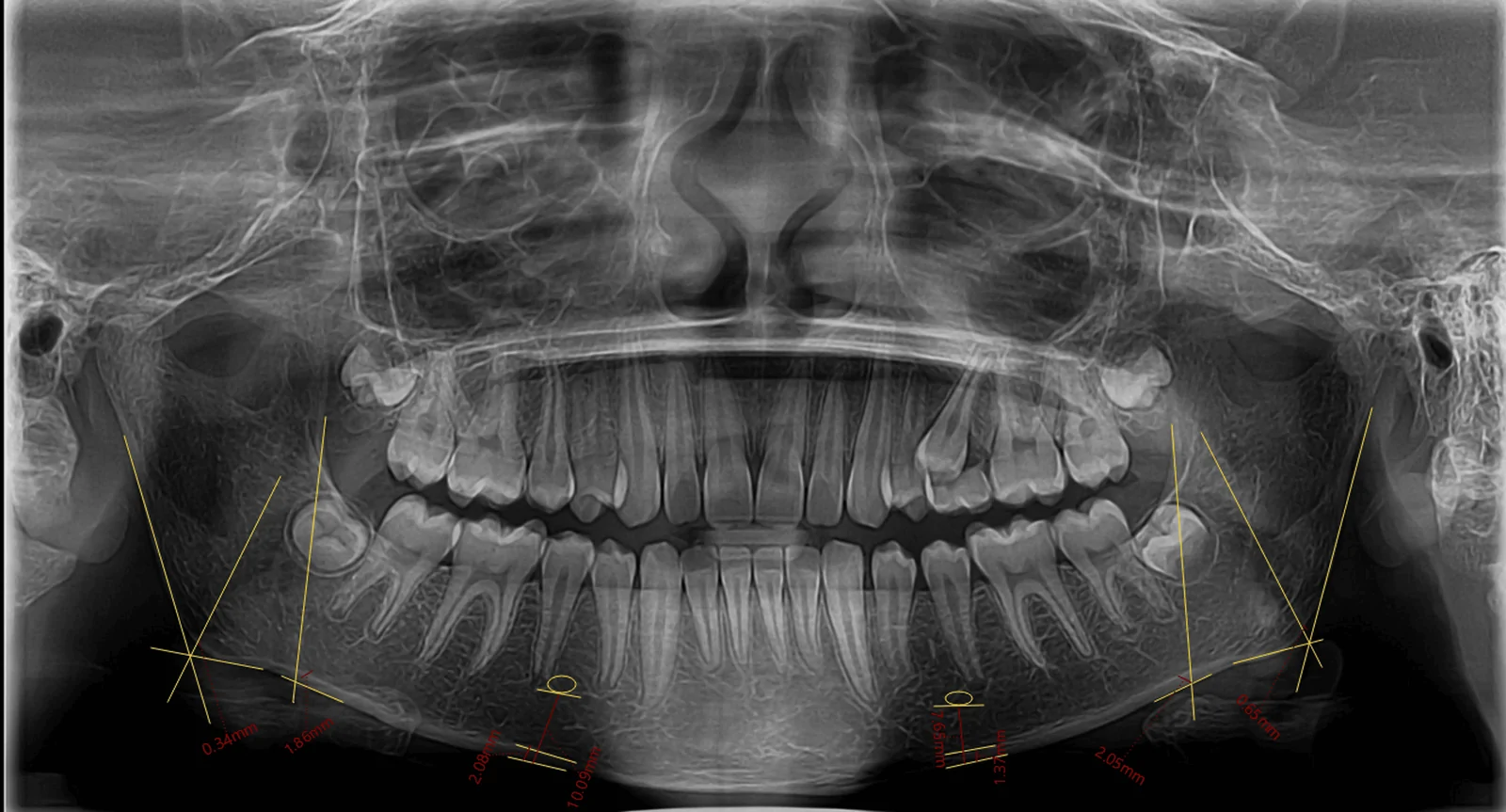
Representative digital panoramic radiograph illustrating bilateral measurement of the quantitative mandibular radio-morphometric indices

Mandibular radio-morphometric assessment was performed on all digital panoramic radiographs using standardized methods. All linear measurements were obtained bilaterally using the digital caliper tool incorporated within the EzDent-i imaging software, and the mean of the right and left measurements was used for statistical analysis (Figures 1, 2).

**Figure 3:**
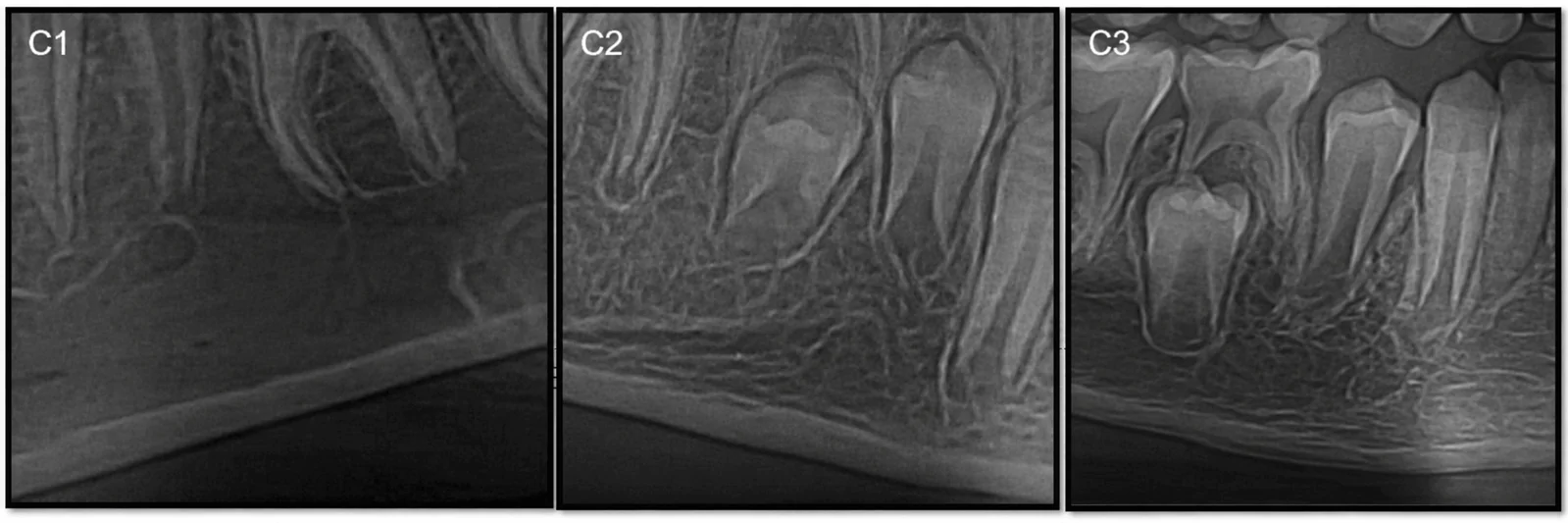
Mandibular Cortical Index (MCI) categories. (C1) Normal cortex. (C2) Mildly to moderately eroded cortex. (C3) Severely eroded cortex.

Standardized mandibular landmarks on each panoramic radiograph were used to derive quantitative indices. The MI was defined as the thickness of the inferior mandibular cortex measured at the level of the mental foramen perpendicular to the lower border of the mandible. The PMI was calculated by dividing MI by the vertical distance from the inferior border of the mandible to the lower border of the mental foramen. The AI was defined as the minimum width of the cortical bone in the region of the antegonion, measured on a line perpendicular to the mandibular base, immediately anterior to the gonial angle. The GI was measured at the angle of the mandible by the bisector of the tangents to the posterior ramus and the inferior border of the mandibular body. The MCI was used for qualitative cortical morphology assessment as described by Klemetti and Kolmakow [10]. C1 had a distinct, uniform, and continuous endosteal outline; C2 had early to moderate erosion with minor concavities or limited remaining cortical layers; and C3 had severe cortical disruption with extensive porosity and an uneven endosteal border (Figure 3).

Examiner calibration and reliability

All radiographic measurements were performed by a single calibrated examiner well-versed in assessing the indices under standardized viewing conditions to minimize measurement bias. To assess intra-observer reliability, 20% of the panoramic radiographs (n = 12) were randomly selected and re-evaluated by the same examiner after a two-week interval without reference to the initial measurements. The intra-observer reliability demonstrated excellent agreement across all quantitative radio-morphometric measurements, with exact Intraclass Correlation Coefficient (ICC) values as follows: MI = 0.94, 95% CI: 0.88-0.97; PMI = 0.91, 95% CI: 0.83-0.95; AI = 0.89, 95% CI: 0.80-0.94; and GI = 0.88, 95% CI: 0.78-0.93.

Statistical analysis

Data analysis was undertaken using IBM SPSS Statistics for Windows, Version 27 (Released 2020; IBM Corp., Armonk, New York). Continuous variables are reported as mean and standard deviation, whereas categorical findings are presented as numbers and percentages. Distributional normality was examined using the Shapiro-Wilk test. Independent-samples t-tests were used to compare quantitative indices between groups, and Pearson’s chi-square test was applied to MCI categories. All analyses were two-sided, and statistical significance was defined as p < 0.05.

## Results

Participant characteristics

A total of 60 children and adolescents aged 8-19 years were included in the study, comprising 30 patients with beta thalassemia major (Group I) and 30 age- and sex-matched healthy controls (Group II). The study population consisted of 25 (41.7%) males and 35 (58.3%) females. No statistically significant differences were observed in age or sex distribution between the two groups (p > 0.05), confirming adequate matching of the study cohorts.

Comparison of quantitative radio-morphometric indices

The comparison of quantitative mandibular radio-morphometric indices between the two groups is presented in Table 1. Patients with beta thalassemia major demonstrated consistently lower mean values for all evaluated mandibular cortical indices than healthy controls.

**Table 1:** Comparison of quantitative mandibular radio-morphometric indices between children and adolescents with beta thalassemia major and healthy controls Values are presented as mean ± standard deviation. Intergroup comparisons were performed using the independent-samples t-test with 58 degrees of freedom. Negative t values indicate lower mean values in cases than in controls. *Statistically significant at p < 0.05. RTMI, right Mental Index; RTPMI, right Panoramic Mandibular Index; RTAGI, right Antegonial Index; RTGI, right Gonial Index; LTMI, left Mental Index; LTPMI, left Panoramic Mandibular Index; LTAGI, left Antegonial Index; LTGI, left Gonial Index.

Variable	Cases (n = 30), Mean ± SD	Controls (n = 30), Mean ± SD	t value	p value
Right Mental Index (RTMI)	1.8210 ± 0.7066	2.2880 ± 0.6066	−2.747	0.008*
Right Panoramic Mandibular Index (RTPMI)	0.1953 ± 0.0583	0.2907 ± 0.0691	−5.779	<0.001*
Right Antegonial Index (RTAGI)	1.7703 ± 0.6398	2.1600 ± 0.5868	−2.459	0.017*
Right Gonial Index (RTGI)	0.5810 ± 0.3742	0.8817 ± 0.3254	−3.321	0.002*
Left Mental Index (LTMI)	1.7097 ± 0.6286	2.5293 ± 0.6980	−4.779	<0.001*
Left Panoramic Mandibular Index (LTPMI)	0.2050 ± 0.0693	0.3277 ± 0.0748	−6.591	<0.001*
Left Antegonial Index (LTAGI)	1.7993 ± 0.6969	2.2630 ± 0.5990	−2.764	0.008*
Left Gonial Index (LTGI)	0.5443 ± 0.3025	0.9003 ± 0.2998	−4.579	<0.001*

On the right side, the MI was significantly lower in the thalassemia group than in the control group (1.8210 ± 0.7066 mm vs. 2.2880 ± 0.6066 mm; t = −2.747, p = 0.008). Similarly, the PMI was significantly reduced in the thalassemia group (0.1953 ± 0.0583) compared with the control group (0.2907 ± 0.0691; t = −5.779, p < 0.001). The AI was also significantly lower in patients with beta thalassemia major than in healthy controls (1.7703 ± 0.6398 mm vs. 2.1600 ± 0.5868 mm; t = −2.459, p = 0.017). Likewise, the GI was significantly reduced in the thalassemia group (0.5810 ± 0.3742 mm) compared with the control group (0.8817 ± 0.3254 mm; t = −3.321, p = 0.002).

Comparable findings were observed on the left side. The MI was significantly lower in patients with beta thalassemia major than in controls (1.7097 ± 0.6286 mm vs. 2.5293 ± 0.6980 mm; t = −4.779, p < 0.001). The PMI was also significantly reduced in the thalassemia group (0.2050 ± 0.0693) compared with the control group (0.3277 ± 0.0748; t = −6.591, p < 0.001). Significant reductions were similarly observed for the AI (1.7993 ± 0.6969 mm vs. 2.2630 ± 0.5990 mm; t = −2.764, p = 0.008) and the GI (0.5443 ± 0.3025 mm vs. 0.9003 ± 0.2998 mm; t = −4.579, p < 0.001).

**Figure 4:**
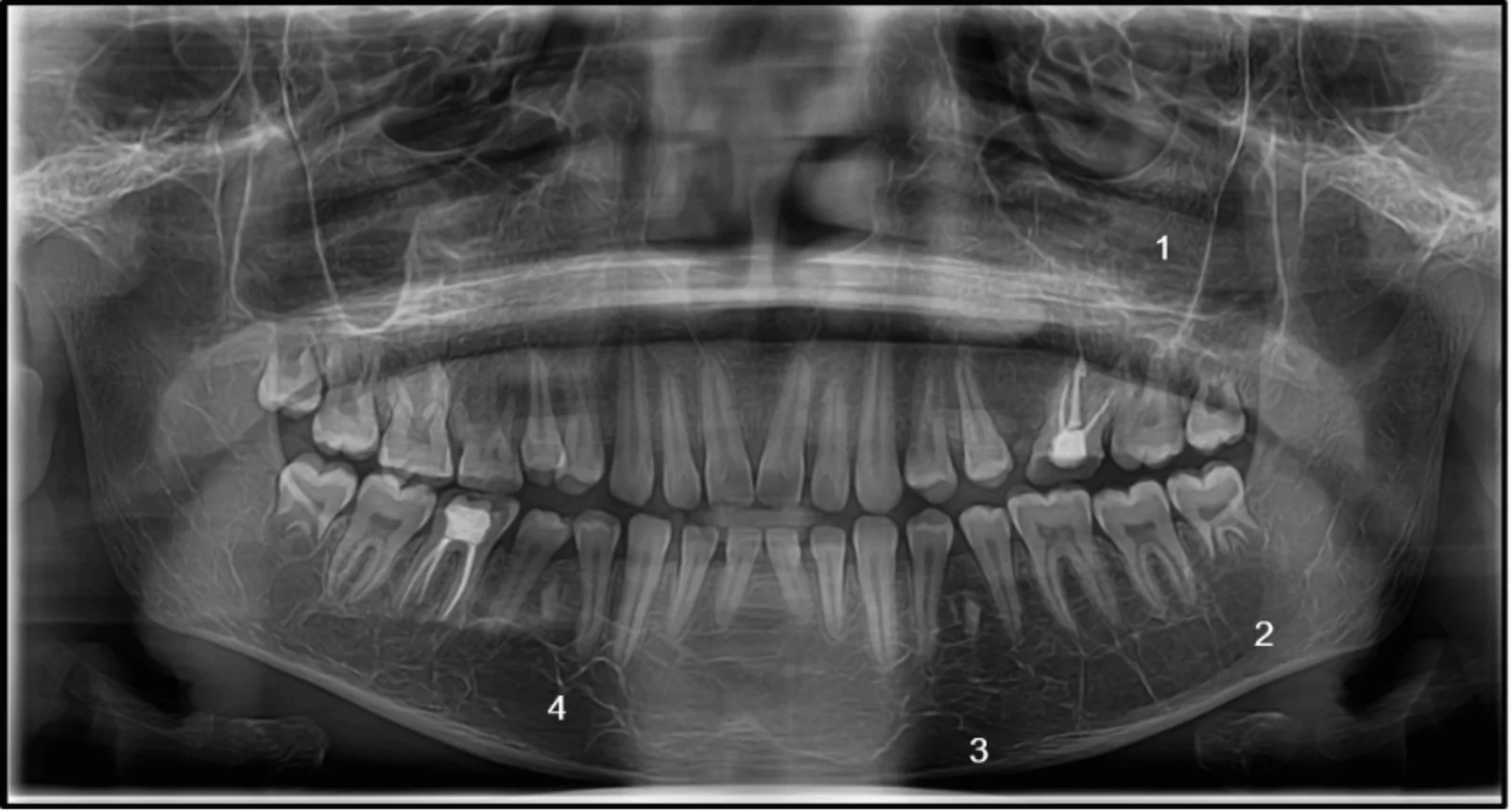
Representative panoramic radiograph of a patient with beta thalassemia major demonstrating characteristic osseous changes. (1) Obliteration of the maxillary sinus, (2) reduced radiographic visibility of the inferior alveolar canal, (3) thinning of the inferior mandibular cortex, and (4) enlargement of the mandibular marrow spaces

Overall, all quantitative mandibular radiomorphometric indices were significantly lower in children and adolescents with beta thalassemia major than in healthy controls. Representative panoramic radiographs demonstrating characteristic osseous changes associated with beta thalassemia major, including obliteration of the maxillary sinus, reduced visibility of the inferior alveolar canal, thinning of the inferior mandibular cortex, and enlargement of the mandibular marrow spaces, are shown in Figure 4.

Distribution of the mandibular cortical index

The distribution of MCI categories between the two groups is presented in Table 2. Significant differences in cortical morphology were observed between patients with beta thalassemia major and healthy controls on both the right and left sides.

**Table 2:** Distribution of Mandibular Cortical Index (MCI) categories between children and adolescents with beta thalassemia major and healthy controls Values are presented as frequencies and percentages. Differences in the distribution of Mandibular Cortical Index categories between cases and controls were assessed using the Pearson chi-square test. *Statistically significant at p < 0.05. MCI, Mandibular Cortical Index; C1, normal cortex; C2, mildly to moderately eroded cortex; C3, severely eroded cortex.

Side	Group	C1, n (%)	C2, n (%)	C3, n (%)	χ²	p value
Right	Cases	5 (16.7)	21 (70.0)	4 (13.3)	15.29	<0.001*
	Controls	19 (63.3)	11 (36.7)	0 (0.0)		
Left	Cases	7 (23.3)	12 (40.0)	11 (36.7)	20.54	<0.001*
	Controls	24 (80.0)	5 (16.7)	1 (3.3)		

On the right side, most healthy controls demonstrated a normal cortical pattern, with 19 participants (63.3%) classified as C1. In contrast, most patients with beta thalassemia major exhibited a mildly to moderately eroded cortex, with 21 participants (70.0%) classified as C2. A severely eroded C3 cortical pattern was observed in four patients (13.3%) with beta thalassemia major but was not observed in the control group. The difference in right-sided MCI distribution between the groups was statistically significant (χ² = 15.29, p < 0.001).

On the left side, C1 morphology predominated among healthy controls, occurring in 24 participants (80.0%). In contrast, C2 and C3 cortical patterns were more frequently observed among patients with beta thalassemia major. Twelve patients (40.0%) exhibited a C2 cortex, and 11 patients (36.7%) demonstrated a C3 cortex, compared with five controls (16.7%) and one control (3.3%), respectively. The difference in left-sided MCI distribution between the groups was also statistically significant (χ² = 20.54, p < 0.001).

These findings demonstrate a greater prevalence of cortical erosion and irregularity among children and adolescents with beta thalassemia major, indicating compromised mandibular cortical integrity compared with healthy controls.

## Discussion

The present study demonstrates that children and adolescents with beta thalassemia major exhibit measurable and statistically significant reductions in mandibular cortical bone mass, clearly detectable through panoramic radio-morphometric analysis. These structural alterations stem primarily from chronic, ineffective erythropoiesis and erythroid marrow hyperplasia [12,13]. As the medullary space expands to compensate for persistent anemia, it exerts continuous mechanical pressure on the surrounding cortical structures, accelerating endosteal bone resorption and progressive thinning of the mandibular cortex [14-16].

The significant bilateral reductions observed across all quantitative indices indicate a generalized pattern of mandibular cortical thinning rather than localized or region-specific resorption. Most notably, left MI values dropped significantly from 2.5293 ± 0.6980 mm in controls to 1.7097 ± 0.6286 mm in thalassemia cases (p < 0.001), alongside a parallel reduction in the PMI (p < 0.001). This widespread cortical compromise is consistent with the systemic nature of marrow expansion in thalassemia, which simultaneously affects multiple craniofacial sites. The present quantitative findings closely align with observations reported by Bayrak et al., who similarly documented marked reductions in MI and PMI values among thalassemia patients relative to healthy controls [17]. Furthermore, studies by Yagmur et al. in pediatric cohorts, as well as work by Hazza’a and Al-Jamal highlighting cortical thinning and altered trabecular architecture at the gonial and antegonial regions, reinforce the consistency of these mandibular morphometric changes across different populations [12,18].

The qualitative findings obtained via the MCI further elucidate the extent of structural degradation within the mandible. A marked shift toward eroded cortical patterns was observed in the thalassemia cohort, where up to 70.0% of cases exhibited C2 mild-to-moderate erosion and over a third demonstrated severe C3 cortical destruction, compared to controls, where normal C1 morphology predominated. This increasing presence of C2 and C3 patterns reflects pronounced endosteal resorption that closely resembles changes seen in metabolic bone conditions such as postmenopausal osteoporosis. This shows early onset of compromised bone quality in transfusion-dependent patients, supporting the broader understanding that reduced bone mineral density and skeletal fragility accumulate rapidly during childhood and adolescence, which is a critical window during which peak bone mass should normally be accrued [19-21]. From a clinical perspective, digital panoramic radiography represents an accessible, cost-effective, and low-radiation imaging modality that can be routinely integrated into pediatric dental care [22-24]. Early identification of mandibular cortical thinning provides pediatric dentists and maxillofacial clinicians with vital insights prior to initiating invasive dental procedures, orthodontic therapy, or surgical extractions, where compromised bone quality could predispose patients to altered healing or structural complications [25,26]. As advocated by Ali et al., integrating routine mandibular radio-morphometric surveillance into comprehensive multidisciplinary management protocols can facilitate timely medical and dental interventions [27].

Limitations

Despite these meaningful findings, certain limitations of the current investigation should be acknowledged. First, the relatively small sample size and single-center nature of the study may limit the generalizability of the findings to broader pediatric and adolescent populations with beta thalassemia major. Although the sample size was determined a priori and was adequate for the planned intergroup comparisons, validation in larger and more diverse cohorts is warranted. Furthermore, because of its cross-sectional design, this study could not track the longitudinal progression of mandibular cortical deterioration over time or assess the direct impact of chelation regimens and transfusion frequency on bone remodeling. Additionally, radio-morphometric measurements were not directly compared with dual-energy X-ray absorptiometry, which is the diagnostic gold standard for bone mineral density, as explored in case-control investigations by Shah et al. [28]. Future prospective multicenter studies involving larger pediatric and adolescent cohorts are recommended to better characterize the progression of thalassemia-induced mandibular changes. Incorporating longitudinal panoramic radio-morphometric tracking alongside dual-energy X-ray absorptiometry analysis, serum ferritin monitoring, and endocrine evaluation will further clarify the relationship between metabolic factors and craniofacial bone remodeling in beta thalassemia major [29]. The results of such studies can also be combined with artificial intelligence technologies and model developments for early diagnosis, treatment monitoring, and prognosis of beta-thalassemia.

## Conclusions

Children and adolescents with beta thalassemia major demonstrated significant reductions in mandibular radio-morphometric indices, including the MI, PMI, AI, and GI, together with a higher prevalence of eroded mandibular cortical patterns compared with healthy controls. These findings indicate compromised mandibular cortical bone quality associated with thalassemia-related skeletal changes. Panoramic radiography, a routinely performed dental imaging modality, may serve as a simple, non-invasive, and cost-effective tool for opportunistic assessment of mandibular bone health and early detection of skeletal alterations in this population. Incorporation of radio-morphometric assessment into routine dental evaluation may facilitate timely referral and multidisciplinary management of children and adolescents with beta thalassemia major. Further multicenter longitudinal studies correlating panoramic radio-morphometric indices with bone mineral density and clinical parameters are necessary to validate their role as imaging biomarkers of skeletal health.
